# Characterization of fecal microbiome in biopsy positive prostate cancer patients

**DOI:** 10.1002/bco2.104

**Published:** 2021-09-07

**Authors:** Ran Katz, Muhamad Abu Ahmed, Ali Safadi, Wasiem Abu Nasra, Alexander Visoki, Michael Huckim, Ibrahim Elias, Meital Nuriel‐Ohayon, Hadar Neuman

**Affiliations:** ^1^ Department of Urology Ziv Medical Center Safed Israel; ^2^ Azrieli Faculty of Medicine Bar‐Ilan University Safed Israel; ^3^ Medical Research Center Ziv Medical Center and Zefat Academic College Safed Israel

**Keywords:** bacterial diversity, fecal flora, microbiome, prostate cancer

## Abstract

**Objectives:**

To characterize the fecal microbiome in newly diagnosed prostate cancer patients.

**Patients and methods:**

Forty‐nine consecutive patients who were referred for trans rectal prostate biopsy were tested. Patients who received antibiotics 3 months prior to the biopsy, patients with history of pelvic irradiation, prostate or colon cancer, inflammatory bowel disease and urinary tract infection were excluded. A rectal swab was obtained just prior to the biopsy, immediately placed in a sterile tube and kept in −80°C. Sequencing was performed for the 16S rRNA 515F + 806R gene fragment using the QIIME2 software. Analytic tests included Beta diversity (Weighted Unifrac, Unweighted Unifrac, Bray‐Curtis), Alpha diversity (Faith, Evenness), Taxa bar plots and PCoA plots.

**Results:**

Forty‐five samples were suitable for analysis with at least 8000 readings per sample. All patients were Caucasian. Twenty patients had prostate cancer and 25 had benign prostates (BPH). Among prostate cancer patients, Gleason Score was 3 + 3 in 11 patients, 3 + 4 in 5, 4 + 3 in 3, and 4 + 4 in 2. There was no statistical difference in demographic parameters between the groups. We identified over 1000 bacterial species, typical for the colonic microbiome. No significant differences in bacterial populations were found between prostate cancer versus benign prostate patients nor between age groups or between subgroups of Gleason or International Society of Uro‐pathology (ISUP) scores.

**Conclusions:**

Although the microbiome has previously been shown to have an impact on the human microenvironment and cancer risk, we could not demonstrate a significant difference between the flora diversity of newly diagnosed prostate cancer patients and BPH patients. Further research into distinct bacterial metabolic pathways may reveal unique risk factors for prostate cancer.

## 1 INTRODUCTION

Prostate cancer is the most common malignancy in men. Recognized risk factors for development of prostate cancer are age, ethnicity, family history of prostate cancer, and genetic mutations. There is accumulating evidence supporting the importance of chronic inflammatory processes, viral and bacterial infections, and nutritional and lifestyle factors on prostate biology.^1^, ^2^, ^3^, ^4^ All these factors also shape the human microbiome and influence the interactions between the gut microbiota and the host, affecting the body's physiology and health.^5^, ^6^, ^7^

The development of new molecular methods for identification and characterization of broad bacterial populations, opened a new era of research and identification of potential relations between bacterial populations and diseases such as diabetes, obesity, and colon cancer.^8^, ^9^, ^10^, ^11^, ^12^

One possible mechanism for malignant transformation is chronic inflammation, leading to pro‐malignant changes in the affected tissue. Therefore, we may assume that certain bacterial populations may contribute to the development of malignancy by inducing a pro inflammatory response or changes in the extra cellular matrix of the prostate.^13^, ^14^ The gut microbiome has an influence on the host immune system, especially affecting regulatory T‐cells, which are involved in anti‐inflammatory processes. Studies with germ‐free rodents colonized with specific microbiota show that commensal microorganisms are required for a functioning immune system.^15^

Various organisms directly infect the prostate, such as enterobacteria or sexually transmitted diseases. The bacteria *Propionbacterium Acne* was directly cultured from prostate cancer lesions and verified by gene amplification techniques.^16^ Analysis of radical prostatectomy specimens using ultra deep pyrosequencing revealed changes in bacterial concentrations as well as subtypes between tumor and benign tissues.^17^ The major sources of these bacteria are the gut and the perineum. Therefore, we conducted a study to evaluate and characterize the bacterial populations in the gut of newly diagnosed, treatment naïve prostate cancer patients and compare them to benign prostatic hypertrophy patients.

## 2 PATIENTS AND METHODS

### 2.1 Recruitment and Sample collection

Recruitment was performed in accordance with the Helsinki declaration and rules of Good Clinical Practice. The study was approved by Ziv Medical Center's IRB (institutional review board) (0094–17‐ZIV) and registered in ClinicalTrials.gov (NCT03709485). We prospectively enrolled patients referred for the first time for trans rectal prostate biopsy due to the suspicion of malignancy. All men were asymptomatic and referred to elevated PSA or PSA rise on follow‐up. We excluded patients who received antibiotics 3 months prior to the biopsy, patients with history of pelvic irradiation, prostate or colon cancer, inflammatory bowel disease, urinary tract infection, chronic liver disease and coeliac. Following informed consent, a rectal swab was obtained just prior to the biopsy, immediately placed in a sterile tube and kept in −80°C. A total of 49 eligible consecutive patients were enrolled. After collecting samples, microbial analysis was performed using one DNA purification kit for the whole series.

### 2.2 Trans rectal biopsy protocol

Patients underwent trans rectal ultrasound guided (B&K flexfocus 800) under local anesthesia using lignocaine gel in the anal canal and periprostatic block injecting 10 ml of lidocaine 2% to the neurovascular bundles of the prostate. We routinely do not give oral antibiotics and do not use suppositories or enemas prior to biopsies. After a rectal swab was obtained, the patients received a single intramuscular injection of Garamycin 240 mg.

### 2.3 DNA extraction and sequencing

Microbial DNA was extracted from rectal swab samples using a PureLink Microbiome DNA purification kit (Invitrogen, Thermo Fisher Scientific, Carlsbad, CA, USA), according to the manufacturer's instructions following a preliminary step of bead‐beating for 2 min. Purified DNA was subjected to PCR amplification using PrimeSTAR Max (TaKaRaClontech, Shiga, Japan) for the variable V4 region (using 515F‐806R barcoded primers) of the 16S rRNA gene. Amplicons were purified using Agencourt AMPure XP magnetic beads (Beckman Coulter, Brea, CA) and subsequently quantified using a Quant‐It PicoGreen double‐stranded DNA (dsDNA) quantitation kit (Invitrogen, Carlsbad, CA). Equimolar amounts of DNA from individual samples were pooled, cleaned by the use of E‐gel (Life Technologies, Carlsbad, CA, USA), and sequenced using the Illumina MiSeq platform at the Genomic Center of the Bar‐Ilan University, at the Azrieli Faculty of Medicine.

### 2.4 16S rRNA gene sequencing and statistical analysis

The sequences were analyzed using QIIME2 software packages. Taxonomy was assigned using the QIIME2 RDP classifier algorithm, at 99% identity to the Greengenes 13.8 reference database. For phylogenetic‐tree‐based analyses, each feature was represented by a single sequence that was aligned using the mafft program. A phylogenetic tree was built with Fast‐Tree and used to estimate the phylogenetic distances between features. Alpha Diversity (Faith's phylogenetic diversity, observed features, Shannon diversity index, and evenness Pielou's index) and Beta diversity (unweighted UniFrac and weighted UniFrac) values were calculated using QIIME2 core‐metrics phylogenetic method. Differences were considered significant for *P* values of 0.05 or below.

## 3 RESULTS

Forty‐five samples were adequate for analysis including 20 patients with prostate cancer and 25 patients with benign prostates. Patients mean age was 68 years (median—67, STD 6.9 interquartile range 62–72). Patient demographics are presented in Table 1. Patients PSA range from 4.5 to 13 nng/ml (median 6.7).

**TABLE 1:** Demographics of 49 men

No.	Age	Ethnicity	Pathology	ISUP score
1	62	Jewish	BPH	
2	71	Jewish	prostate cancer Gleason 3 + 4 (7)	2
3	67	Jewish	prostate cancer Gleason 4 + 3 (7)	3
4	61	Arab	prostate cancer Gleason 3 + 3 (6)	1
5	79	Jewish	BPH	
6	72	Jewish	prostate cancer Gleason 3 + 3 (6)	1
7	81	Jewish	BPH	
8	65	Jewish	BPH	
9	64	Jewish	prostate cancer Glean 3 + 3 (6)	1
10	57	Jewish	BPH	
11	72	Jewish	prostate cancer Gleason 3 + 3 (6)	1
12	59	Jewish	BPH	
13	58	Jewish	BPH	
14	70	Jewish	prostate cancer Gleason 3 + 3 (6)	1
15	68	Jewish	BPH	
16	60	Jewish	prostate cancer Gleason 4 + 3 (7)	3
17	66	Jewish	BPH	
18	60	Jewish	BPH	
19	66	Arab	BPH	
20	72	Jewish	prostate cancer Gleason 3 + 4 (7)	2
21	71	Jewish	prostate cancer Gleason 3 + 3 (6)	1
22	67	Jewish	prostate cancer Gleason 3 + 3 (6)	1
23	71	Jewish	prostate cancer Gleason 3 + 3 (6)	1
24	68	Jewish	BPH	
25	79	Jewish	BPH	
26	56	Jewish	BPH	
27	72	Arab	prostate cancer Gleason 3 + 3 (6)	1
28	64	Jewish	prostate cancer Gleason 4 + 4 (8)	4
29	73	Jewish	prostate cancer Gleason 3 + 3 (6)	1
30	71	Arab	BPH	
31	66		BPH	
32	73		BPH	
33	68		prostate cancer Gleason 4 + 4 (8)	4
34	74		prostate cancer Gleason 3 + 4 (7)	2
35	67		prostate cancer Gleason 3 + 3(6)	1
36	60		prostate cancer Gleason 3 + 3 (6)	1
37	67		BPH	
38	67		BPH	
39	77		BPH	
40	74		prostate cancer Gleason 3 + 4 (7)	2
41	62		prostate cancer Gleason 3 + 3 (6)	1
42	70		BPH	
43	79		BPH	
44	81		prostate cancer Gleason 3 + 4 (7)	2
45	64		prostate cancer Gleason 3 + 3 (6)	1
46	54		BPH	
47	57		BPH	
48	79		prostate cancer Gleason 4 + 3 (7)	3
49	61		BPH	

Twenty patients were diagnosed with prostate cancer and 25 had benign prostates. There was no statistical difference between the groups in terms of age (mean age 70 and 67 years respectively, *p* = 0.27).

Of the 20 prostate cancer patients, 10 patients had a Gleason score of 3 + 3, five patients had a Gleason score of 3 + 4, three patients had 4 + 3, and two patients had 4 + 4.

**FIGURE 1:**
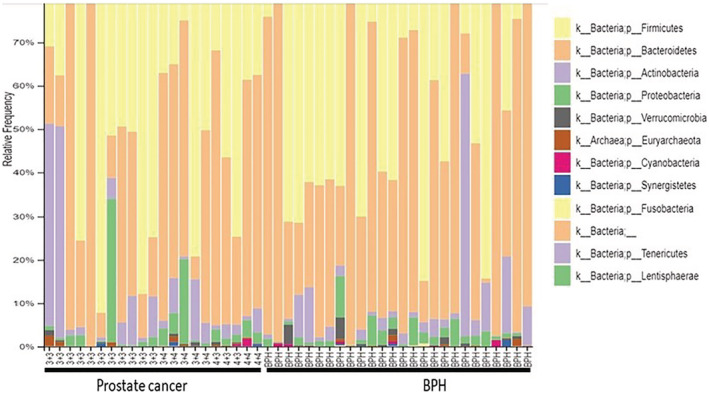
Bar plots representing distribution of bacterial phyla in 45 rectal swab samples

Sequencing was performed for the 16S rRNA 515F + 806R gene fragment using the QIIME2 software. More than 8000 readings were obtained from each sequence. Over 1000 bacterial species were identified, typical for normal fecal microbiome, with Firmicutes, Bacteroidetes, and Actinobacteria phyla being most abundant (Figure 1).

**FIGURE 2:**
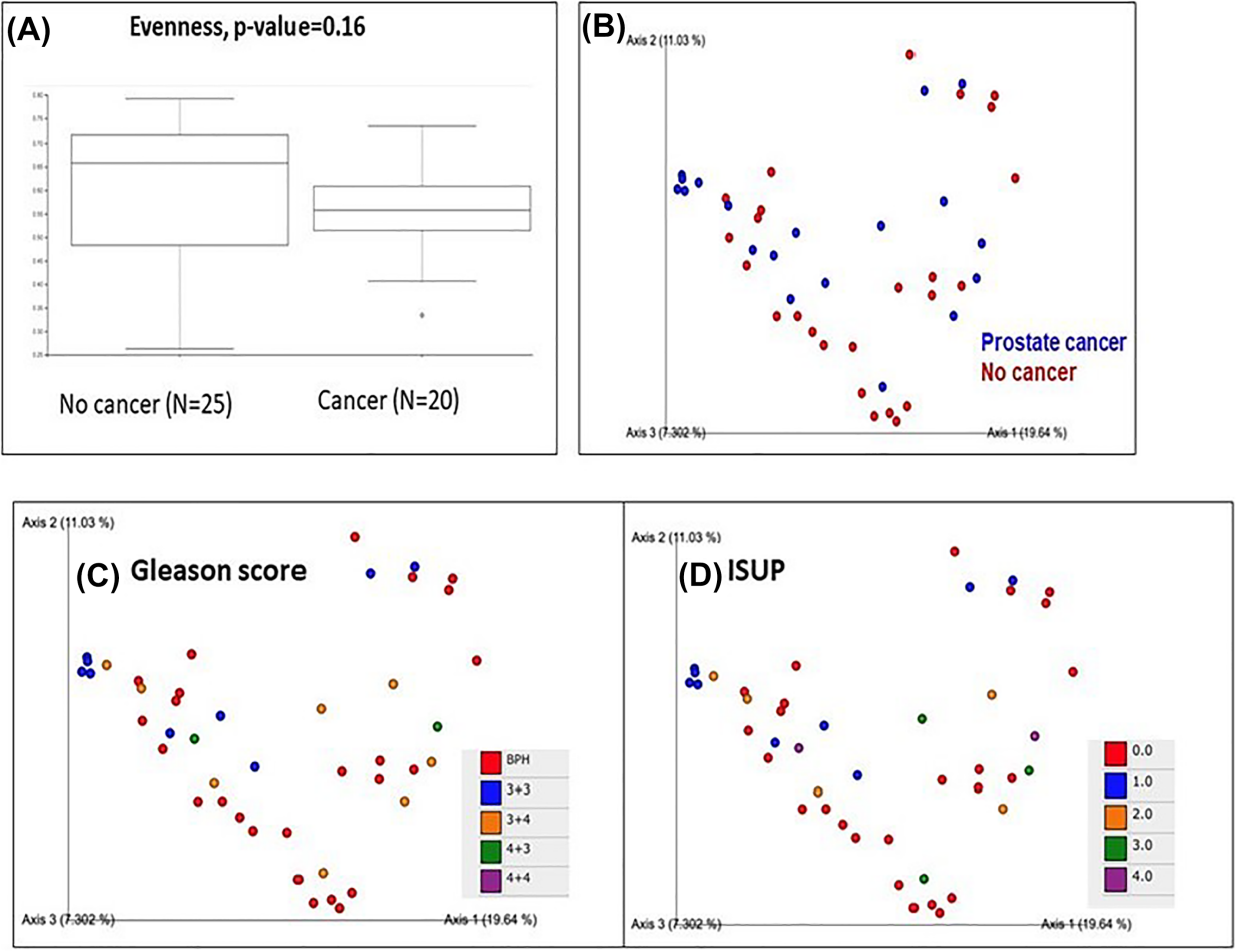
(A) Box plot of alpha diversity (Evenness) displaying no significant differences between cancerous and benign samples (*p* = 0.16). (B) Principal coordinates analysis (PcoA) plot based on Bray–Curtis dissimilarities of microbiotas from cancerous (blue) and benign (red) samples. (C) PcoA of microbiotas from cancerous samples according to Gleason score. (D) PcoA of microbiotas from cancerous samples according to International Society of Uro‐pathology (ISUP) score

Looking at the diversity of bacterial populations, no difference was found between cancerous and benign samples (Evenness test, *p* = 0.16). A box‐plot is presented in Figure 2A. Similarly, we observed no group clustering or significant differences in beta diversity as seen by the Principal coordinates analysis (PcoA) plot (Figure 2B).

We looked for differences in bacterial distribution patterns between the various Gleason scores or the International Society of Uro‐pathology (ISUP) scores and found no significant differences. Principal coordinates analysis (PcoA) plots are presented in Figure 2C,D, respectively.

**FIGURE 3:**
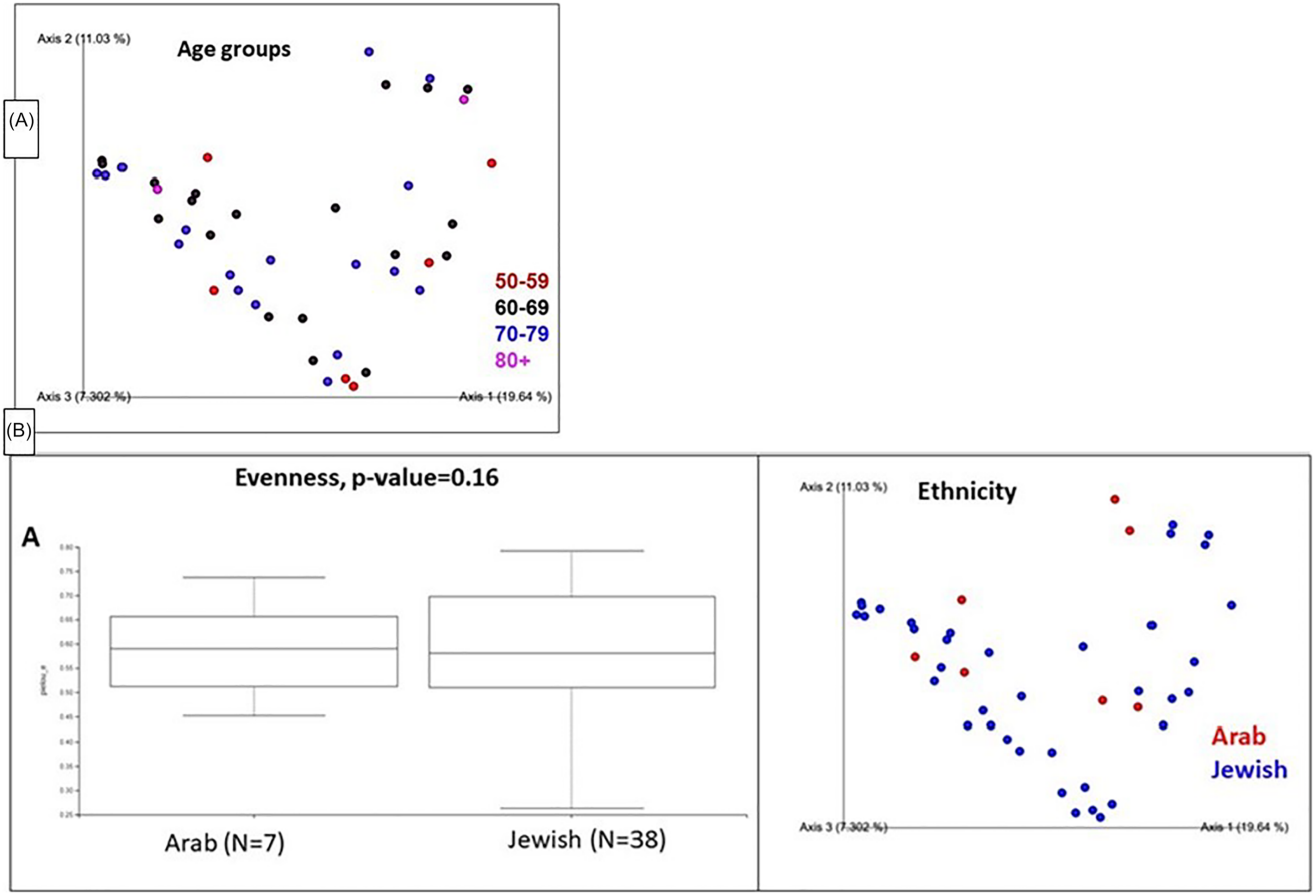
(A) Principal coordinates analysis (PcoA) plot based on Bray–Curtis dissimilarities of microbiotas of the study population stratified to age groups. (B) Left—Box plot of alpha diversity (Evenness) displaying no significant differences between ethnic groups (*p* = 0.16). Right—Principal coordinates analysis (PcoA) plot based on Bray—Curtis dissimilarities of microbiotas from Jewish (blue) and Arab (red) samples

When stratifying the research population to 10 years age groups, we did not find any correlation between age and specific bacterial species patterns. A principal coordinates analysis (PcoA) plot is presented in Figure 3A.

The population was also analyzed according to ethnicity. Thirty‐eight patients were Jewish and seven were Arabs. No difference in microbial populations was noted between the groups (Figure 3B).

## 4 DISCUSSION

Prostate cancer is the most common malignancy in men and second leading cause of men mortality in the US. Therefore, research is mainly focused on early detection of prostate cancer and identifying risk factors for aggressive or recurrent disease, such as serum biomarkers, genetic alterations and ethnicity.

The human microbiome encompasses millions of active organisms that influence and shape the body's microenvironment in various pathways, such as direct infection and transformation, induction of an inflammatory response and release of cytokines, immune and autoimmune responses and metabolic pathways. Additionally, the microbiotas synthesize and supplement the body with metabolites that it cannot produce.^5^, ^6^, ^7^

One possible connection between the gut microbiome and prostate malignancy may be supported by the fact that the prostate is often infected by colonic bacteria, mainly *Escherichia coli*, and that acute and chronic inflammatory changes are often seen in benign and cancerous prostate specimens.^18^

Cavaretta et al. examined 16 radical prostatectomy specimens and analyzed them for the presence as well as concentration of bacteria within the tumor, around it and in benign sections of the prostate. They noticed an abundance of various species. They found a significant higher concentration of streptoccoci and staphylococcus species in the tumor and peritumor tissue compared to benign regions of the specimen. This study was limited, however, by the small cohort and lack of a control group.^17^

The histological diagnosis of prostate cancer relies on prostate biopsies, which are mainly performed trans‐rectally under ultrasound guidance. The biopsies themselves infect the prostate and clinical prostatitis was reported in 1%–2.5% of patients undergoing trans‐rectal biopsies in various series. Trans‐perineal prostate biopsies allow sterile biopsies yet often require general anesthesia or sedation and are by far less common.^19^ Targeted prostate biopsies, based on fusion images of MRI scans of the prostate, are becoming more common due to the improved rate of detection of clinically significant prostate cancer. Yet they lead to clustering of biopsies in tumor regions which might influence bacterial concentrations in the tissue. For future studies, trans‐perineal MRI fusion targeted biopsies may be the ideal setup.

Our cohort was rather small and included 45 patients, yet it was very homogenous as all participants were Caucasian males. They were all cancer naïve and did not have a previous prostate biopsy. We excluded patients with previous urinary tract infection and other conditions that might affect the gut microbiome such as inflammatory bowel disease of bowel or pelvic irradiation, liver disease and Celiac disease. We did not look specifically into factors such as specific diet, or the use of proton pump inhibitors (PPI's) for peptic disease which may also influence the microbiome. As seen from the figures, over 1000 bacteria species were identified, typical for colonic microbiome, and representing an adequate sample, and the abundance of bacterial species compensates for the rather small number of patients. No differences in bacterial population were found between prostate cancers versus benign prostates, as well as between age groups.

We used a systematic biopsy template taking six random biopsies from each prostatic lobe. Eventually, we found 20 patients with prostate cancer and 25 controls. There was no statistical difference in demographic parameters between the groups.

We used the rectal swab just prior to the biopsy in order to collect the bacterial culture. While stool samples may improve the harvesting of material, they significantly reduce compliance.^20^ We obtained adequate material for analysis in 45 of 50 patients.

We further analyzed the results according to the pathological Gleason score or the ISUP score and found no significant difference between the sub‐groups.

The study has limitations: The sample size was rather small and therefore underpowered. We compensated for that buy performing a prospective study with a homogenous cohort and by the wide diversity of fecal microbiota in the samples. Selection bias might ensue due to fact that these were referred patients, and not a random sample of the population. Also, cancers may have been missed as no MRI was used in the evaluation process of the patients.

Despite the lack of difference between the bacterial populations, there remains a possibility for differences in the microbial activity such as their metabolic pathways. The gut microbiome contributes to the metabolism of glycans, amino acids, and xenobiotics.^21^ Liss et al. investigated 133 rectal swabs taken from patients referred for prostate biopsies. They found that the fecal microbiome of men undergoing prostate biopsy is similar between cancer and noncancerous groups. Yet men without prostate cancer had a significantly higher percentage of bacteria producing folate and B vitamins.^22^ Our data support their findings and emphasizes the need for further studies regarding the possible differences between various bacterial species such as their metabolic activity.

## 5 CONCLUSIONS

In this study we examined prostate biopsy naïve patients and did not demonstrate a difference between the gut microbiota diversity of prostate cancer versus BPH patients. This suggests that the microbiome composition is not a relevant biomarker for prostate cancer diagnosis. However, functional differences in microbiome parameters were not tested in this work. Therefore, microbial metabolic pathway analysis may still potentially reveal risk factors for prostate cancer development.

## CONFLICT OF INTEREST

None.

## AUTHOR CONTRIBUTION

Dr. Katz—head of project, writing and data analysis. Dr. Abu Ahmed, Abu Nasra, Visoky, Huckim, and Elias—clinical trial, obtaining clinical samples. Dr. Nuriel Ohayon and Dr. Neuman—Microbiom analysis, data analysis, and proofreading.
